# Why We Sleep: A Hypothesis for an Ultimate or Evolutionary Origin for Sleep and Other Physiological Rhythms

**DOI:** 10.5334/jcr.189

**Published:** 2020-03-30

**Authors:** Andrew S. Freiberg

**Affiliations:** 1 Penn State Health, Department of Pediatrics, Penn State Hershey Medical Center, Hershey, PA, US

**Keywords:** sleep, evolution, proximate cause, ultimate cause, physiology, adaptive theory, restorative theory

## Abstract

Although sleep is ubiquitous, its evolutionary purpose remains elusive. Though every species of animal, as well as many plants sleep, theories of its origin are purely physiological, e.g. to conserve energy, make repairs or to consolidate learning. An evolutionary reason for sleep would answer one of biology’s fundamental unanswered questions. When environmental conditions change on a periodic basis (winter/summer, day/night) organisms must somehow confront the change or else be less able to compete in either niche. Seasonal adaptation includes the migration of birds, changes in honeybee physiology and winter abscission in plants. Diurnal adaptation must be more rapid, forcing changes in behavior in addition to physiology. Since organisms must exist in both environments, evolution has created a way to force a change in behavior, in effect creating “different” organisms (one awake, one asleep) adapted separately to two distinct niches. We sleep to allow evolving into two competing niches. The physiology of sleep forces a change to a different state for the second niche. The physiological needs for sleep are mechanisms that have evolved to achieve this goal.

It is ‘one of the last great biological mysteries’ [1] that although sleep is ubiquitous and essential [2], we have yet to determine its true evolutionary purpose [3]. Even though every species of animal, as well as many plants, go to sleep every day, a satisfactory answer to one of biology’s fundamental questions is lacking [4,5,6,7].

We know a lot about the physiology of sleep, with more being learned every day. We know what happens during sleep, and what happens when we or other animals are deprived of it [1,4,8,9]. What we still do not know is why sleep exists in the first place.

What Allen Rechtschaffen wrote in 1998 is still true [10]:

A number of sleep theories have been put forth and fluctuations in biological patterns have been measured during sleep, but the function of sleep is not yet understood. Sleep can be understood as fulfilling many different functions but intuition suggests there is one essential function. The discovery of this function will open an important door to the understanding of biological processes.

From a biological and evolutionary standpoint, sleep theories can be categorized into those that consider proximate or ultimate causes [11]. Proximate causes explore sleep as a process – the physiology of sleep and sleep deprivation, which is the focus of nearly all current research. Ultimate or evolutionary causes have been proposed, broadly categorized as ***restorative*** or ***adaptive*** theories [12].

Restorative, or recuperative theories hypothesize that sleep serves one or more of the following functions: to rest and repair [13,14]; to consolidate what we have learned while we were awake [7,15,16,17,18]; to dream [19]; to enhance the immune response [15,19,20,21]; to avoid the serious detrimental effects of deprivation [1,4,9,22]; to detoxify [23].

In fact, all of these do occur during sleep, and have been found to be important, even essential functions of the complicated sleep process, i.e. proximate causes. However, as evolutionary causes, for the most part these theories have fallen apart under scrutiny [10]. For example, energy expenditure is not significantly lower during sleep, and if sleep were important to learning, why is it present in lower orders of animals, single celled organisms, and even in many plants? In fact, a general criticism of restorative theories is that they apply only to a limited set of tissues or species. Dreams may be an essential function of sleep in humans and other primates or even all mammals [19], but extending that function to earthworms and daylilies is difficult to imagine.

Circadian rhythms are ubiquitous across nature as nearly every organism studied, including bacteria, algae, fungi, plants and animals, all exhibit physiological cycles that are near to 24 hours in length, even when uncoupled from the daily light/dark cycle [24]. Furthermore all cells of all organisms studied so far have such rhythms [24]. Yet the more that is known about these rhythms at the cellular and molecular level, the less such explanations as detoxification, immune enhancement, memory consolidation and tissue repair make sense as underlying causes for the presence of sleep.

Intuitively, an organism that could stay awake for 24 hours would survive better, with the ability to be productive around the clock, competing for precious resources. A sleeping animal is not engaging in productive activities and is vulnerable to predation. Adaptive theories state that sleep is a way of keeping us away from dangerous conditions such as predators. I believe adaptive theories may be partially correct, but they do not go far enough. Discovering the true *evolutionary* function of sleep, i.e. the reason sleep adds to the *fitness* of an organism, would explain ‘Why we sleep.’

I propose a subtle yet profound explanation to the question of why we and every animal species on earth sleep. This theory in no way invalidates or takes the place of any of the current explanations of the functions and purposes of sleep, the proximate causes. Rather, it relegates these functions into physiological tactics that serve a larger evolutionary strategy – the true ultimate purpose of sleep.

From an evolutionary standpoint, all structure and function (i.e. physiology) exists because it confers some survival advantage. Since physiology includes behavior, sleep must confer an evolutionary advantage as a behavior. The central question concerning sleep is thus: what evolutionary advantage could sleep possibly provide?

All organisms occupy a niche, and the better adapted to that niche, the more ‘fit’ and the more likely that organism will reproduce, passing on the characteristics that fit that particular niche. While we may simplistically think of each organism occupying a single niche, realistically nearly all occupy at least two. Daytime and nighttime are different and distinct niches, creating an evolutionary push and pull that would make a perfect ‘fit’ impossible. Evolutionarily, being forced to evolve into two separate niches at the same time forces an organism to develop structures and functions that fit neither fully.

We and nearly every other species on earth must navigate an environment of approximately half light and half dark. Since other organisms change in response to that cycle, there are also different *biological* environments that exist between night and day, further enforcing the differences between the two niches.

The physiology of every organism is driven by evolutionary pressure from its environment. Millions of years before there were animals, plants, or even DNA on the Earth, there were two niches: day and night. The instant life began these two very different environments were waiting for it, at nearly every spot on Earth. Early life and all subsequent life was forced to confront a dilemma of how to adapt to these two rapidly alternating niches. Being awake day and night forces an organism to adapt to both light and dark, as well as to the different organisms populating each, and to do so would compromise its ability to perform well in either. Since the day/night schedule has been around since before there was life on Earth it would be ingrained in all but the most sequestered organisms. The daily cycle is so constant, and at such a high frequency, repeating every single day, that it is impossible to keep up (i.e. circumnavigating the earth every day) so the only alternative is to change physiology to match. The day/night problem therefore created a powerful, pervasive and inescapable selection pressure. Adaptation to these two niches must therefore lie deep within our DNA, as in fact, we find a circadian clock functioning in all cells of all organisms.

Evolution quickly devised a way for organisms, particularly animals to change physiology each day – they go to sleep. It is as if evolution were saying ‘Pick one, either day or night, and optimize your structure, function and behavior for that environment, then get away from the one you did not choose, to avoid attempting to optimize to both.’

Thought of in this way, sleep is not only controlled by, but acts a powerful guardian of, circadian rhythms.

To be sure, we do of course exist in both the day and night niches, but we and nearly every other animal species have consequently developed a way to change our physiology and behavior sufficiently that we become, not literally, but in a sense two distinct organisms, one adapted to each niche, rather than a single physiology adapted to both, and therefore poorly adapted to either. The “asleep” organism is not living its own life but exists in order to deliver at wake time the shared organism in the best possible shape for the wake journey of foraging, hunting, or working.

The *adaptive* theory states that animals sleep to avoid danger [12,25]. For example, animals that are active during the day are vulnerable to predators at night, and therefore sleep to avoid being hunted and eaten. This theory explains why such an animal would need to *hide* during the night, but not why they sleep, although it makes sense that an animal might want to conserve energy while hiding away and not actively foraging for food. The adaptive theory goes part way to the truth, but instead of simply hiding from danger and conserving energy while doing so, the physiological need for sleep not only hides these animals from danger during the night, but ensures they will stay hidden on evolutionary time scales.

Sleep is important and we can’t live without it – on the surface because of some need to repair or replenish our bodies. More importantly, deep down, the need to repair or replenish is a trick our DNA plays on us to keep us from being up at night. Because we need sleep every day we never have the opportunity to adapt to the darkness, which would compromise our ability to survive during the day. In the adaptive theory, we hide from the danger of darkness; in this new theory we hide from the danger of *adapting* to the darkness.

Of course there is no absolute division between diurnal and nocturnal species. Across species, the quantity and timing of sleep patterns varies greatly [20]. While some animals are clearly one or the other, there are those that are active for short periods both day and night, or active at twilight, and others for which some members are diurnal and others nocturnal, and in some cases even a single individual can change from one to the other based on environmental conditions [26]. Like other traits sleep can be modified experimentally [27,28,29]. Yet the fundamental need for sleep itself is non-negotiable from an evolutionary standpoint. All of these organisms, even those that are intermittently active day and night still have functioning circadian clocks and all of them still require sleep [2]. The variety of sleep patterns illustrates that whatever the ultimate cause for sleep is, it does not require a single prolonged period of rest, as in humans, and is obviously not dependent on any particular sleep architecture. Even animals that are in a niche that allows day and night activity have not given up the need for sleep, showing how fundamentally important it is. The need for sleep is in all organisms’ DNA, but does not govern how that need is satisfied; that physiology evolves with each species as the environment still has a lot to say. All animals, for example need to retain the ability for arousal during sleep, but to different amounts and in different ways depending on their circumstances. Unihemispheric sleep is another example of how sleep has been adapted to specific needs [19,30,31,32]. Evolution has allowed for a variety of ways to spend the day and night, within which the ultimate need for sleep persists, yet has no direct control over.

Learning is an essential process of the human species, and sleep is essential to the consolidation of memories. Yet, it is not sleep, but rather sleep *architecture* that has co-evolved with learning and other recuperative processes to its present state, to optimize these processes for each species.

One advantage this theory has over others is that it can be broadly applied to all species, from unicellular organisms all the way to *Homo sapiens*.

This theory can be extended to other biological rhythms: There are of course two examples of periodic and predictable changes in environment, the diurnal and the seasonal. Marcus Hall introduces the term “chronophilia” to explain how life has adapted to the periodic environmental changes occurring on Earth due to its rotation and axial tilt [33]. While his article is mostly concerned with human activity, nearly all life on Earth is subjected to the same temporal constraints.

In temperate and many tropical climates there are profound differences between the conditions of summer and winter, chiefly hot/cold and wet/dry, and few organisms can adapt well to both. Triggered by changes in light, temperature and moisture, animals and plants, by well understood biochemical processes, change their biology to survive in the two climates [34,35,36]. Deciduous trees lose their leaves and go into a hibernation mode to survive the winter, while evergreen trees change the chemistry of their internal fluids to keep from freezing [37]. Many animals go through similar changes, such as hibernating or changing color to blend in to different surroundings. These examples illustrate that when an organism is faced with periodic and predictable environmental changes, in order to survive in both environments, it must periodically change either 1) its biology; or 2) its surroundings; or 3) both.

Migrating birds, mammals and insects effectively change their environment seasonally. Why do some birds fly south for the winter? Not simply to stay warm. They fly south to prevent genetic adaptation to the winter, which would compromise their physiology and fitness in the summer. Similarly some animals hibernate not just to avoid the cold winter, but to keep from adapting to it. Honeybee workers in winter live months rather than weeks to avoid having to raise brood during the cold winter [35].

Why do some trees lose their leaves in the fall? The mechanisms and evolutionary advantages of this process, known as abscission, are well understood [38,39]. However, I hypothesize that the evolutionary reasons behind abscission go beyond a simple ‘cost benefit’ analysis. Leaves are not useful in the dark and cold of winter; so many plants lose them to prevent the creation of an “all season” leaf, which would be an arboreal bad idea. It takes a lot of energy and material to make a leaf; trees would not give them up without a very good reason. Evergreen trees of course do not shed, but fundamentally change the physiology of their needles in the winter [37].

Humans today have the technology to control their environment to a large extent. If we so choose we could live in a world of constant light or constant darkness. The reason we don’t is that we sleep better in darkness, the night niche. This behaviour keeps us in sync with the sunlight. Our circadian clocks, are, by the process of entrainment, constantly resetting to changes that occur with the seasons, our schedules, or with daylight savings time. When we travel across time zones our circadian clocks become significantly out of sync, but still manage to use the local zeitgebers to reset so that we keep our daylight physiology and night physiology in tune with the local day/night schedule.

Adaptive theories of sleep have never really taken hold, while research on recuperative, physiological theories has exploded, possibly due to the fact that recuperative theories are so much more testable than adaptive. This new theory may open a window for experimentation. While difficult, my theory is testable, both by experimental and investigative methods.

If we had evolved on a planet with no day night cycles would we still sleep? Unfortunately we are a long way from being able to perform that experiment now, if ever. For seasonal variation the experiment has been done: Do organisms that live in constant climates need to change physiology twice each year? Of course not – why would they need to?

The bottom line and the answer to the question: “Why do we sleep?”

Sleep is a tactic evolution uses to serve a greater strategy: We sleep to allow adaptation into two competing niches. The need for sleep forces us to change our physiology into a different state for the second niche. All the other answers (why we NEED sleep) are but mechanisms to achieve this goal.
